# Mews at the time of ICU discharge is associated with outcome

**DOI:** 10.1186/2197-425X-3-S1-A136

**Published:** 2015-10-01

**Authors:** IA Meynaar, P Huber, AE van den Berg, J Vermeulen, K Toorenburg, P Melief, R Baak

**Affiliations:** HagaZiekenhuis, ICU, The Hague, Netherlands

## Introduction

The objective of this study was to see if the modified early warning score (MEWS) on discharge from the ICU to the ward is associated with outcome.

## Patients and Methods

The study was done in the HagaZiekenhuis, a 600-bed teaching hospital in The Hague, Netherlands with all specialties available including cardiac surgery and neurosurgery. The hospital has a 16-bed adult ICU and an ICU based medical emergency team (MET). A MEWS is used throughout the hospital to monitor patients' vital signs and a score of 3 points or more is the threshold to alert the patients' doctor and the MET if necessary. As of 2013 the MEWS was to be recorded in the ICU discharge notes of all patients discharged from the ICU to the nursing wards of the hospital. For all consecutive patients admitted to the ICU in 2013 and discharged to the ward we collected baseline characteristics, outcome data and MEWS on discharge to study the association between outcome as measured by

1. post-ICU hospital mortality,
2. post ICU length-of-stay and
3. ICU readmission rate.

## Results

During the study period, 1297 individual patients were admitted to the ICU. One-hundred and fifty four patients died in the ICU, 75 patients were discharged towards home or another hospital and in 355 patients the MEWS was not recorded in the discharge notes, leaving us with a study population of 713 patients who were discharged from the ICU tot the ward with MEWS available.

1. Chi square test
2. t-Test
3. Mann Whitney U test
4. in hospital survivors (n = 680)

## Conclusions

In patients discharged from the ICU to the ward, a high MEWS at the time of ICU discharge is associated with a significantly higher post-ICU mortality rate, a significantly higher ICU readmission rate and, in hospital survivors, a significantly increased hospital length-of -stay (LOS) after ICU discharge.

**Table 1:** Basic characteristics.

	All patients (n = 713)	MEWS on ICU discharge < = 2 (n = 663)	MEWS on ICU discharge > = 3 (n = 50)	P
Male	497 (69.7%)	459 (69.2%)	38 (76.0%)	ns (1)
Age	64.7 (13.6)	64.8 (13.6)	64.1 (13.3)	ns (2)
Apache IV on ICU admission	59.0 (25.1)	57.7 (24.5)	75.9 (26.8)	< 0.001 (2)
LOS ICU	0.9 (0.8-2.0)	0.9 (0.8-1.9)	2.3 (0.9-5.7)	< 0.001 (3)

**Table 2:** Outcome data.

	All patients	MEWS on ICU discharge < = 2	MEWS on ICU discharge > = 3	p
Hospital mortality	33/733 (4.6%)	22/663 (3.3%)	11/50 (22.0%)	< 0.001 (1)
LOS after ICU discharge (4)	5.2 (4.1-9.8)	5.2 (4.1-9.2)	8.2 (5.2-14.2)	0.005 (3)
ICU readmission	45/713 (6.3%)	37/626 (5.6%)	8/50 (16.0%)	0.01 (1)

